# Urinary metabolites and fatigue in a population-based metabolomics study: an exploratory analysis

**DOI:** 10.1007/s11306-026-02432-6

**Published:** 2026-04-29

**Authors:** Annika Kneipp, Inge Kirchberger, Dennis Freuer, Christine Meisinger, Jakob Linseisen

**Affiliations:** Institute of Epidemiology, Faculty of Medicine, University of Augsburg, 86156 Augsburg, Germany

**Keywords:** Fatigue, Urinary metabolites, Metabolomics, Biomarkers

## Abstract

**Introduction:**

Nearly every fifth adult person suffers from chronic fatigue, but its etiology and related mechanisms are poorly understood. Metabolomics data offer new possibilities for identifying metabolites related to disease etiology.

**Objectives:**

This study aimed to investigate the association between urinary metabolites and fatigue in the general population.

**Methods:**

Fatigue severity was assessed using the Fatigue Assessment Scale (FAS). 51 urinary metabolites were quantified via ¹H nuclear magnetic resonance (¹H-NMR). Multivariable linear regression models adjusted for possible confounders were performed on data of 570 participants of the *Metabolism*,* Nutrition and Immune System in Augsburg* (MEIA) study to explore the associations between urinary metabolites and fatigue.

**Results:**

Four urinary metabolites showed significant associations with fatigue, namely hypoxanthine (β = 0.932, 95% CI 0.168–1.697, *p* = 0.017), 3-hydroxyhippurate (β = 0.213, 95% CI 0.003–0.424, *p* = 0.047), dimethylamine (β = 0.756, 95% CI 0.255–1.257, *p* = 0.003), and trimethylamine-N-oxide (β = 0.088, 95% CI 0.002–0.174, *p* = 0.045). Stratified analyses showed that the association of hypoxanthine was limited to individuals with obesity (BMI ≥ 30 kg/m^2^; β = 3.186, 95% CI 1.742–4.629, *p* < 0.001). After correction for multiple testing (false discovery rate), none of these associations remained statistically significant.

**Conclusion:**

Urinary metabolites related to purine degradation and gut microbial metabolism may reflect fatigue-related biological processes, demonstrating the potential of urinary metabolomics to identify biochemical alterations and possible mechanisms of fatigue pathophysiology.

**Supplementary Information:**

The online version contains supplementary material available at 10.1007/s11306-026-02432-6.

## Introduction

Fatigue is a medical condition that affects cognitive, physical, or emotional domains (Skau et al., 2021). Individuals affected by fatigue commonly describe it as a pervasive sense of mental or physical exhaustion, reduced energy, and difficulty initiating or sustaining activities, often accompanied by a prolonged need for recovery (Chaudhuri & Behan, 2004; Fukuda et al., 1994). Fatigue can occur as a symptom across a broad range of medical conditions, including malignant disorders, diabetes, chronic obstructive pulmonary disease, and cardiovascular diseases. In contrast, it may also present as a distinct clinical entity with established diagnostic criteria, as in myalgic encephalomyelitis/chronic fatigue syndrome (ME/CFS) (Park et al., 2024). Importantly, fatigue in the general population represents a broader and more heterogeneous construct that is not equivalent to ME/CFS, as it lacks specific diagnostic criteria and may arise from a wide range of physiological, psychological, and environmental factors.

In the general population, 18% of individuals report chronic fatigue for over six months (Pawlikowska et al., 1994). Its debilitating and far-reaching consequences, including significant reductions in functional capacity and overall quality of life, highlight the need to understand its underlying pathophysiology to identify targets for treatment (Park et al., 2024). However, consistent fatigue-related biochemical alterations have not been clearly established (Jin et al., 2009).

Metabolomics may help identify such biochemical changes, as it reflects integrated genetic, environmental, dietary, and microbial influences on human physiology, thereby providing a closer representation of an individual’s current functional state than genomic data alone (Fernie et al., 2004).

Metabolomics has proven valuable in studying complex diseases by revealing disease-specific metabolic patterns and offering insights relevant to diagnosis and treatment. This approach has already yielded important findings in conditions comparable in complexity to fatigue, including fibromyalgia, chronic widespread pain, and irritable bowel syndrome (Freidin et al., 2018; Hackshaw et al., 2019; Ponnusamy et al., 2011).

Metabolomic studies specifically investigating fatigue are limited and have largely focused on clinical samples, mainly individuals with ME/CFS (Abujrais et al., 2024; Armstrong et al., 2015; Nagy-Szakal et al., 2018; Naviaux et al., 2016). Existing studies are primarily based on blood analyses and have reported alterations in metabolites related to energy, lipid, nucleotide, and amino acid metabolism. However, studies examining fatigue in the general population remain scarce (Freidin et al., 2018; Nozaki et al., 2009), and urinary metabolomic data are still limited (Armstrong et al., 2015; Nozaki et al., 2009).

To address this gap, we conducted an explorative urinary metabolomic analysis in a general population sample using NMR spectroscopy-derived data. Our objective was to identify metabolites associated with fatigue and thereby generate hypotheses on potential underlying biological pathways for future research.

## Materials & methods

### Study sample

Data from the *Metabolism*,* Nutrition and Immune System in Augsburg* (MEIA) study, a population-based study conducted by the Chair of Epidemiology at the University Hospital Augsburg, were used for this research. The MEIA study is a population-based study designed to investigate the interplay between nutrition, the immune system, and health, and to provide a basis for future longitudinal research. The target sample size was set at a minimum of 500 participants based on overall study objectives and feasibility considerations. A total of 594 participants, aged 18 to 75 years and living in the region of Augsburg, Germany, were randomly selected and examined between April 2021 and July 2023. Individuals unable to provide informed consent were excluded.

For the present analyses, 24 participants were excluded due to missing data on the Fatigue Assessment Scale (*n* = 13) or urinary metabolite measurements (*n* = 11), resulting in a final sample size of 570 individuals.

### Data collection

Data were collected at the study center of the Chair of Epidemiology, University Hospital Augsburg. Participants completed a standardized, computer-assisted questionnaire assessing demographic characteristics, health conditions, lifestyle factors, and fatigue symptoms, among other variables. Collected information included sex, age, educational level, smoking status, alcohol consumption, and physical activity. Self-reported health conditions comprised depression, myocardial infarction, stroke, diabetes, history of COVID-19 and cancer.

In addition, participants completed a series of physical, cognitive, and anthropometric examinations (including body weight and height) during their study center visits. Dietary intake data were collected by means of the web tool myfood24 (Dietary Assessment Ltd, Leeds, United Kingdom) over the months following the study center visit (Carter et al., 2015). Bio-specimens, including fasting venous blood, spot urine, and fecal samples, were obtained from all participants. Blood specimens were analyzed for standard clinical parameters such as lipid profiles and inflammatory markers. All bio-samples were pre-processed under standardized conditions and subsequently stored at −80 °C at the Chair of Epidemiology for future research. For the present analysis, spot urine samples were used for metabolomic profiling.

### Fatigue assessment

Fatigue was evaluated using the Fatigue Assessment Scale (FAS), a widely used self-report questionnaire designed to measure fatigue in the general population (Michielsen et al., 2003). The instrument comprises 10 items, each rated on a five-point Likert scale ranging from “never” to “always”, yielding a total score between 10 and 50. According to established cut-off values, a score below 22 indicates no fatigue, scores from 22 to 35 reflect a moderate level of fatigue, and scores above 35 represent a high level of fatigue (Lynch et al., 2007).

### Metabolomics analysis in urine samples

20 mL spot urine samples were collected after an overnight fast (≥ 12 h), centrifuged, aliquoted, and stored at −80 °C until analysis. Urinary metabolomic profiling (50 metabolites + creatinine) was performed by Nightingale Health Ltd. using a high-throughput NMR platform with automated total-line-shape fitting (Bizzarri et al., 2023; Mutter et al., 2022).

To account for dilution effects, metabolite concentrations were normalized to urinary creatinine and subsequently scaled by a factor of 100 to facilitate interpretation (Li et al., 2022). All values below the limit of detection were replaced with half the lowest detected concentration for the respective analyte in this study, corresponding to the analytical detection limit.

Samples were thawed, centrifuged, mixed with phosphate buffer, and analyzed using a 600 MHz Bruker AVANCEIIIHD NMR spectrometer with automated sample changer and cryoprobe at 298 K (Mutter et al., 2022).

Spectra were acquired using standard water-suppressed measurements (32 scans/sample, 5.1 s recycle time), followed by automated total-line-shape fitting after piece-wise spectral alignment. Metabolite assignment was based on published chemical shift and J-coupling data and confirmed with spiking and calibrated concentrations using standard addition (Mutter et al., 2022).

Quality control procedures included monitoring of biomarker distributions compared to reference data, quantification success rates, and quality control tag rates. These measures were performed by the laboratory conducting the analysis (Nightingale Inc.).

### Confounders

Potential confounding factors were selected based on prior literature and included demographic characteristics, lifestyle factors, and fatigue-related health-conditions. Variables comprised age (years), sex, body mass index (BMI, kg/m^2^), calculated from measured body weight and height, and educational attainment, according to the International Standard Classification of Education (ISCED levels 1–3 and 4–6) (OECD, 1999). Smoking status was classified as “never,” “former,” or “current.” Alcohol consumption was assessed using the Alcohol Use Disorders Identification Test (AUDIT) and divided into four categories using sex-specific cut-offs (maximum score 12): “low” (men 0–3, women 0–2), “moderate” (men 4–5, women 3–5), “high” (both sexes 6–7), and “severe” (both sexes 8–12) (Saunders et al., 1993). Physical activity was evaluated using the European Health Interview Survey-Physical Activity Questionnaire (EHIS-PAQ), which captures frequency, duration, and intensity of activity (Finger et al., 2015). Participants were subsequently classified into four physical activity levels: “sedentary,” “low active,” “active,” and “very active” (Gerrior et al., 2006).

Fatigue-related health conditions were incorporated in a second model and included depression, major cardiovascular diseases (CVD; stroke or myocardial infarction), diabetes, self-reported history of COVID-19 and cancer.

### Statistical analysis

Patient characteristics and urinary metabolites were summarized as absolute and relative frequencies for categorical variables and as median and 25th and 75th quantiles for continuous variables.

For group comparisons, the FAS score was dichotomized at a cut-off of 22 (score < 22: no fatigue; ≥ 22: fatigue) (Lynch et al., 2007). Differences in baseline characteristics between groups were assessed using Chi-square tests for categorical variables and Mann-Whitney U tests for continuous variables. Corresponding *p*-values < 0.05 were considered statistically significant.

Of the 51 quantified urinary metabolites, creatinine was used solely for normalization and was therefore not included as an independent variable in the statistical analyses. Additionally, four metabolites (3-hydroxyisovalerate, ethanol, 2-furoylglycine, and glucose) were excluded due to extreme skewness, low variability, or zero inflation. This exclusion was based on inspection of the metabolite distributions using boxplots and histograms, resulting in 46 metabolites included in subsequent statistical analyses.

To investigate the association between each urinary metabolite (exposure) and fatigue score (continuous outcome), multiple linear regression models were performed. All assumptions of linear regression models were ensured by visual inspection of Q-Q plots for residual normality and Cook’s distance to identify influential observations. The linearity between continuous covariables and the outcome was confirmed using polynomial terms of degree two.

The main regression models were adjusted for potential confounders, including age, sex, BMI, education level, physical activity level, smoking status and alcohol consumption. A second model further included fatigue-related health conditions as covariates: depression, CVD, diabetes, COVID-19 and cancer.

Potential interactions between age, sex, and BMI with each urinary metabolite were tested by including corresponding interaction terms in the regression models and evaluating their statistical significance using *p*-values. According to the results, stratified analyses were performed by BMI category: underweight/normal weight (BMI < 25 kg/m^2^), overweight (25 ≤ BMI < 30 kg/m^2^), and obese (BMI ≥ 30 kg/m^2^) (Zierle-Ghosh & Jan, 2025).

To account for multiple testing, *p*-values were adjusted using the Benjamini–Hochberg false discovery rate (FDR) procedure.

All statistical analyses were performed using IBM SPSS Statistics, version 28 (IBM Corp., Armonk, NY, USA).

## Results

### Descriptive data

Table 1 summarizes the sociodemographic, lifestyle, and clinical characteristics of the study population, stratified by sex or fatigue status. Of the 570 participants included in the analysis, 320 were women and 250 were men. The median age was 49 years (34.0; 60.0), with no significant differences by sex (*p* = 0.637) or fatigue status (*p* = 0.339). Approximately half of the participants fell into the healthiest category for each lifestyle factor, including 49.4% reporting low alcohol consumption, 50.0% that have never smoked, and 50.7% classified as active or very active. Sex differences were observed primarily in alcohol consumption patterns and BMI, with men more frequently reporting risky alcohol consumption and having higher median BMI values compared to women (*p* < 0.001). 

Overall, 76.7% of participants reported no fatigue, 22.5% were classified as having mild fatigue and 0.9% as having severe fatigue. Men had a median FAS score of 18 (15; 21), while women had a slightly higher median score of 19 (16; 22). However, the prevalence of mild or severe fatigue did not differ significantly between sexes (*p* = 0.068). 

Participants with fatigue were more likely to report lower physical activity (*p* = 0.027) and a history of depression (*p* < 0.001) or prior COVID-19 (*p* = 0.018) than those without fatigue. Specifically, 18.2% of individuals with fatigue reported a history of depression compared to only 4.3% of those without fatigue. A smaller proportion of fatigued participants were classified as active or very active (42.0%) compared to participants without fatigue (53.3%).

**Fig. 1:**
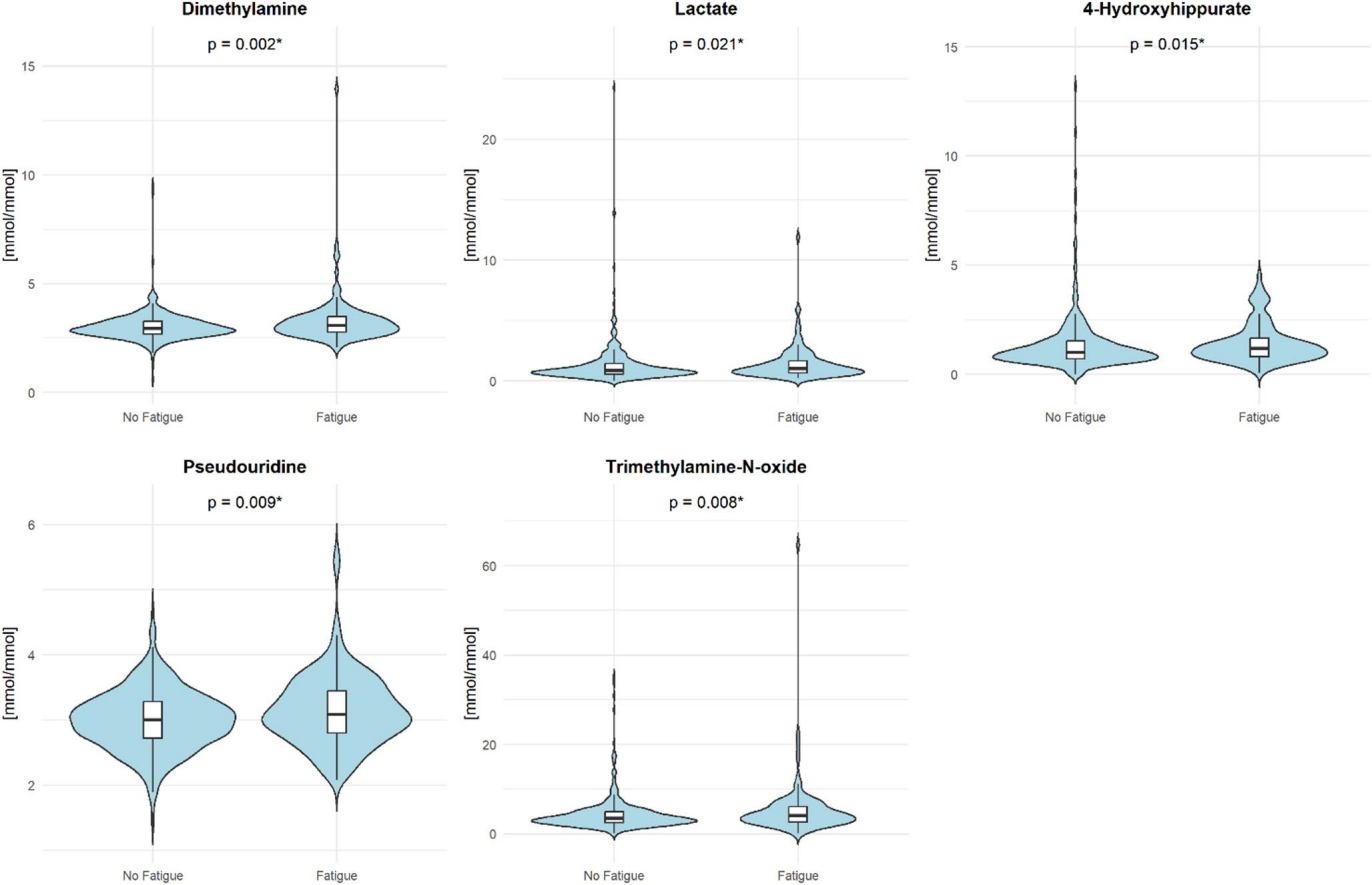
Violin plots with embedded boxplots showing urinary metabolite concentrations [mmol/mmol creatinine x 100] by fatigue status. Group differences were assessed using the Mann–Whitney U test; p-values are displayed with * indicating *p* < 0.05

The distribution of selected urinary metabolites by fatigue status is shown in Fig. 1. The metabolites displayed correspond to those showing significant differences between groups. Individuals with fatigue exhibited higher concentrations of dimethylamine, lactate, 4-hydroxyhippurate, pseudouridine, and trimethylamine-N-oxide compared to those without fatigue. Detailed results on all urinary metabolite concentrations, stratified by sex and fatigue status, are provided in Table 2 and Supplementary Table S1, respectively. Significant sex-related differences were observed for a range of metabolites, with women generally exhibiting higher concentrations than men. This pattern was evident for several metabolites, including acetate, arabinose, cis-aconitate, citrate, dimethylamine, ethanolamine, formate, glucose, glycine, hippurate and hypoxanthine, among others.

**Table 1:** Baseline characteristics of participants of the population-based MEIA study, stratified by fatigue status

	Total (*n* = 570)	Female (*n* = 320)	Male (*n* = 250)	*p*-values	No fatigue (*n* = 437)	Fatigue (*n* = 133)	*p*-values	*N* ^c^
*Median (Q25, Q75)*								
Age [y]	49.0 (34.0; 60.0)	49.0 (34.0; 58.0)	50.0 (34.0: 61.0)	0.637^a^	49.0 (34.0; 61.0)	48.0 (34.3; 57.0)	0.339^a^	570
BMI [kg/m^2^]	25.7 (22.7; 29.1)	24.5 (21,3; 28.5)	26.8 (24.5; 29.6)	< 0.001^a^	25.6 (22.7; 29.0)	26.2 (22.8; 29.8)	0.420^a^	569
Fatigue-score	19 (15; 21)	19 (16; 22)	18 (15; 21)	0.067^a^	17 (14; 20)	24 (23; 27)	< 0.001^a^	570
*n (%)*								
Fatigue level								
No (0–21)	437 (76.7%)	234 (73.1%)	203 (81.2%)	0.068^b^				570
Mild (22–34)	128 (22.5%)	83 (25.9%)	45 (18.0%)					
Severe (> 34)	5 (0.9%)	3 (0.9%)	2 (0.8%)					
Risky alcohol consumption								
Low	280 (49.4%)	163 (51.4%)	117 (46.8%)	< 0.001^b^	208 (47.9%)	72 (54.1%)	0.525^b^	567
Moderate	212 (37.4%)	137 (43.2%)	75 (30.0%)		165 (38.0%)	47 (35.3%)		
High	53 (9.3%)	12 (3.8%)	41 (16.4%)		44 (10.1%)	9 (6.8%)		
Severe	22 (3.9%)	5 (1.6%)	17 (6.8%)		17 (3.9%)	5 (3.8%)		
Smoking status								
Current	93 (16.3%)	48 (15.0%)	45 (18.0%)	0.301^b^	72 (16.5%)	21 (15.8%)	0.213^b^	570
Never	285 (50.0%)	169 (52.8%)	116 (46.4%)		226 (51.7%)	59 (44.4%)		
Previous	192 (33.7%)	103 (32.2%)	89 (35.6%)		139 (31.8%)	53 (39.8%)		
Physical activity								
Sedentary	111 (19.6%)	69 (21.7%)	42 (16.9%)	0.173^b^	74 (17.0%)	37 (28.2%)	0.027^b^	566
Low active	168 (29.7%)	100 (31.4%)	68 (27.4%)		129 (29.7%)	39 (29.8%)		
Active	160 (28.3%)	86 (27.0%)	74 (29.8%)		128 (29.4%)	32 (24.4%)		
Very active	127 (22.4%)	63 (19.8%)	64 (25.8%)		104 (23.9%)	23 (17.6%)		
Education level								
ISCED 1–3	116 (20.4%)	65 (20.3%)	51 (20.4%)	0.508^b^	85 (19.5%)	31 (23.3%)	0.494^b^	570
ISCED 4–6	454 (79.6%)	255 (79.7%)	199 (79.6%)		352 (80.5%)	102 (76.7%)		
Depression								
Yes	43 (7.6%)	25 (7.8%)	18 (7.2%)	0.440^b^	19 (4.3%)	24 (18.2%)	< 0.001^b^	569
No	524 (92.1%)	293 (91.6%)	231 (92.8%)		418 (95.7%)	106 (80.3%)		
Unknown	2 (0.4%)	2 (0.6%)	0 (0.0%)		0 (0.0%)	2 (1.5%)		
CVD								
Yes	19 (3.3%)	11 (3.4%)	8 (3.2%)	0.882^b^	14 (3.2%)	5 (3.8%)	0.743^b^	569
No	550 (96.7%)	309 (96.6%)	241 (96.8%)		423 (96.8%)	127 (96.2%)		
Diabetes								
No diabetes	464 (81.4%)	266 (83.1%)	198 (79.2%)	0.570^b^	358 (81.9%)	106 (79.7%)	0.380^b^	570
Prediabetes	64 (11.2%)	32 (10%)	32 (12.8%)		45 (10.3%)	19 (14.3%)		
Diabetes	42 (7.4%)	22 (6.9%)	20 (8.0%)		34 (7.8%)	8 (6.0%)		
COVID-19								
Yes	186 (32.7%)	113 (35.3%)	73 (29.3%)	0.222^b^	137 (31.4%)	49 (37.1%)	0.018^b^	569
No	379 (66.6%)	204 (63.8%)	175 (70.3%)		299 (68.4%)	80 (60.6%)		
Unknown	4 (0.7%)	3 (0.9%)	1 (0.4%)		1 (0.2%)	3 (2.3%)		
Cancer								
Yes	41 (7.2%)	25 (7.8%)	16 (6.4%)	0.526^b^	31 (7.1%)	10 (7.6%)	0.851^b^	569
No	528 (92.8%)	295 (92.2%)	233 (93.6%)		406 (92.9%)	122 (92.4%)		

^a^Mann-Whitney-U-test

^b^Chi^2^ -test

^c^Number of cases with valid information

**Table 2:** Creatinine-corrected urinary metabolite concentrations [mmol/mmol creatinine x 100] in the total study population and stratified by fatigue status

	Total (*n* = 570)	No fatigue (*n* = 437)	Fatigue (*n* = 133)	*p*-values^a^
Acetate	0.463 (0.261; 0.794)	0.448 (0.259; 0.757)	0.526 (0.303; 0.806)	0.246
Alanine	1.728 (1.323; 2.215)	1.728 (1.315; 2.205)	1.764 (1.340; 2.278)	0.729
Allantoin	0.543 (0.270; 0.962)	0.543 (0.270; 0.961)	0.517 (0.286;0.954)	0.845
2-hydroxyisobutyrate	0.494 (0.401; 0.592)	0.485 (0.402; 0.579)	0.520 (0.400; 0.618)	0.078
Arabinose	0.444 (0.296; 0.645)	0.436 (0.296; 0.632)	0.480 (0.319; 0.676)	0.232
3-aminoisobutyrate	0.498 (0.160; 1.249)	0.503 (0.159; 1.295)	0.461 (0.165; 1.092)	0.611
3-hydroxyisobutyrate	0.681 (0.522; 0.872)	0.680 (0.521; 0.872)	0.681 (0.524; 0.863)	0.725
Cis-aconitate	1.726 (1.386; 2.230)	1.703 (1.386; 2.210)	1.791 (1.340; 3.1108)	0.138
Citrate	19.103 (11.615; 28.874)	18.935 (11.527; 27.947)	20.690 (14.846; 31.108)	0.276
Dimethylamine	2.970 (2.705; 3.330)	2.932 (2.694; 3.275)	3.074 (2.778; 3.484)	0.002*
4-deoxyerythronic acid	0.685 (0.529; 0.911)	0.678 (0.527; 0.897)	0.713 (0.547; 0.940)	0.274
4-deoxythreonate	2.094 (1.554; 2.715)	2.144 (1.608; 2.720)	1.933 (1.441; 2.667)	0.054
Ethanolamine	3.992 (3.014; 5.060)	3.974 (3.032; 5.002)	4.111 (2.989; 5.236)	0.638
Formate	1.509 (0.996; 1.989)	1.460 (0.973; 1.990)	1.556 (1.040; 1.975)	0.382
Glutamine	2.029 (1.135; 3.161)	2.036 (1.061; 3.146)	2.022 (1.289; 3.181)	0.471
Glycine	8.007 (5.458; 11.543)	7.932 (5.422; 11.539)	8.575 (5.866; 11.219)	0.278
Glycolic acid	3.882 (2.749; 5.242)	3.777 (2.709; 5.195)	4.127 (2.835; 5.283)	0.221
Hippurate	23.768 (14.685; 38.276)	23.349 (15.065; 37.321)	23.233 (13.005; 40.761)	0.660
3-(3-hydroxyphenyl)-3-hydroxypropionic acid	1.412 (0.469; 2.891)	1.422 (0.490; 2.800)	1.409 (0.283; 3.281)	0.813
Hypoxanthine	0.824 (0.592; 1.125)	0.811 (0.591; 1.085)	0.902 (0.621; 1.177)	0.063
Isoleucine	0.086 (0.056; 0.137)	0.086 (0.056; 0.132)	0.092 (0.056; 0.148)	0.402
Indoxyl sulfate	2.528 (1.792; 3.478)	2.483 (1.739; 3.454)	2.683 (2.029; 3.590)	0.165
Lactate	0.893 (0.583; 1.523)	0.561 (0.377; 0.559)	0.643 (1.685; 2.045)	0.021*
Leucine	0.172 (0.134; 0.224)	0.171 (0.134; 0.218)	0.174 (0.135; 0.245)	0.180
Mannitol	1.045 (0.405; 3.199)	1.023 (0.414; 3.172)	1.261 (0.386; 3.186)	0.806
3-hydroxyhippurate	1.409 (0.721; 2.817)	1.408 (0.721; 2.778)	1.436 (0.729; 2.816)	0.648
1-methylnicotinamide	0.619 (0.457; 0.843)	0.614 (0.455; 0.826)	0.642 (0.475; 0.915)	0.339
Pyroglutamate	2.252 (1.907; 2.667)	2.246 (1.908; 2.650)	2.262 (1.918; 2.698)	0.664
4-hydroxyhippurate	1.047 (0.731; 1.557)	1.011 (0.707; 1.528)	1.186 (0.800; 1.649)	0.015*
Propylene glycol	0.362 (0.187; 0.611)	0.350 (0.183; 0.613)	0.411 (0.173; 0.598)	0.962
Proline betaine	0.630 (0.297; 1.491)	0.630 (0.321; 1.551)	0.612 (0.360; 1.453)	0.684
Pseudouridine	3.024 (2.738; 3.321)	3.001 (2.723; 3.284)	3.088 (2.799; 3.446)	0.009*
Quinic acid	2.215 (1.028; 3.772)	2.218 (0.950; 3.568)	2.190 (1.153; 4.115)	0.373
Sucrose	0.050 (0.000; 0.227)	0.047 (0.000; 0.202)	0.071 (0.000; 0.318)	0.169
Trans-aconitate	0.431 (0.321; 0.546)	0.427 (0.317; 0.541)	0.439 (0.336; 0.562)	0.367
Taurine	2.912 (0.571; 6.524)	2.898 (0.592; 6.434)	3.097 (0.379; 6.962)	0.994
Threonine	0.583 (0.390; 0.833)	0.583 (0.391; 0.836)	0.586 (0.398; 0.815)	0.787
Trimethylamine-N-oxide	3.546 (2.480; 5.201)	3.423 (2.462; 4.981)	4.033 (2.595; 6.093)	0.008*
Trigonelline	2.851 (1.393; 4.668)	2.820 (1.412; 4.658)	3.191 (1.382; 4.591)	0.657
Tryptophan	0.559 (0.405; 0.736)	0.558 (0.422; 0.750)	0.561 (0.423; 0.795)	0.527
Tyrosine	0.926 (0.649; 1.274)	0.918 (0.615; 1.278)	0.958 (0.733; 1.250)	0.229
Uracil	0.518 (0.389; 0.677)	0.514 (0.385; 0.671)	0.543 (0.412; 0.710)	0.105
Urea	3211.916 (2387.135; 4121.429)	3180.077 (2332.427; 4084.925)	3313.750 (2429.201; 4165.769)	0.369
Valine	0.216 (0.166; 0.273)	0.210 (0.166; 0.272)	0.227 (0.174; 0.274)	0.310
Xanthosine	0.887 (0.783; 1.019)	0.876 (0.174; 1.013)	0.915 (0.807; 1.051)	0.064
Xylose	0.600 (0.353; 0.799)	0.602 (0.378; 1.196)	0.592 (0.334; 0.818)	0.938

*p < 0.05

^a^Mann-Whitney-U-Test

### Associations between urinary metabolites and fatigue

**Fig. 2:**
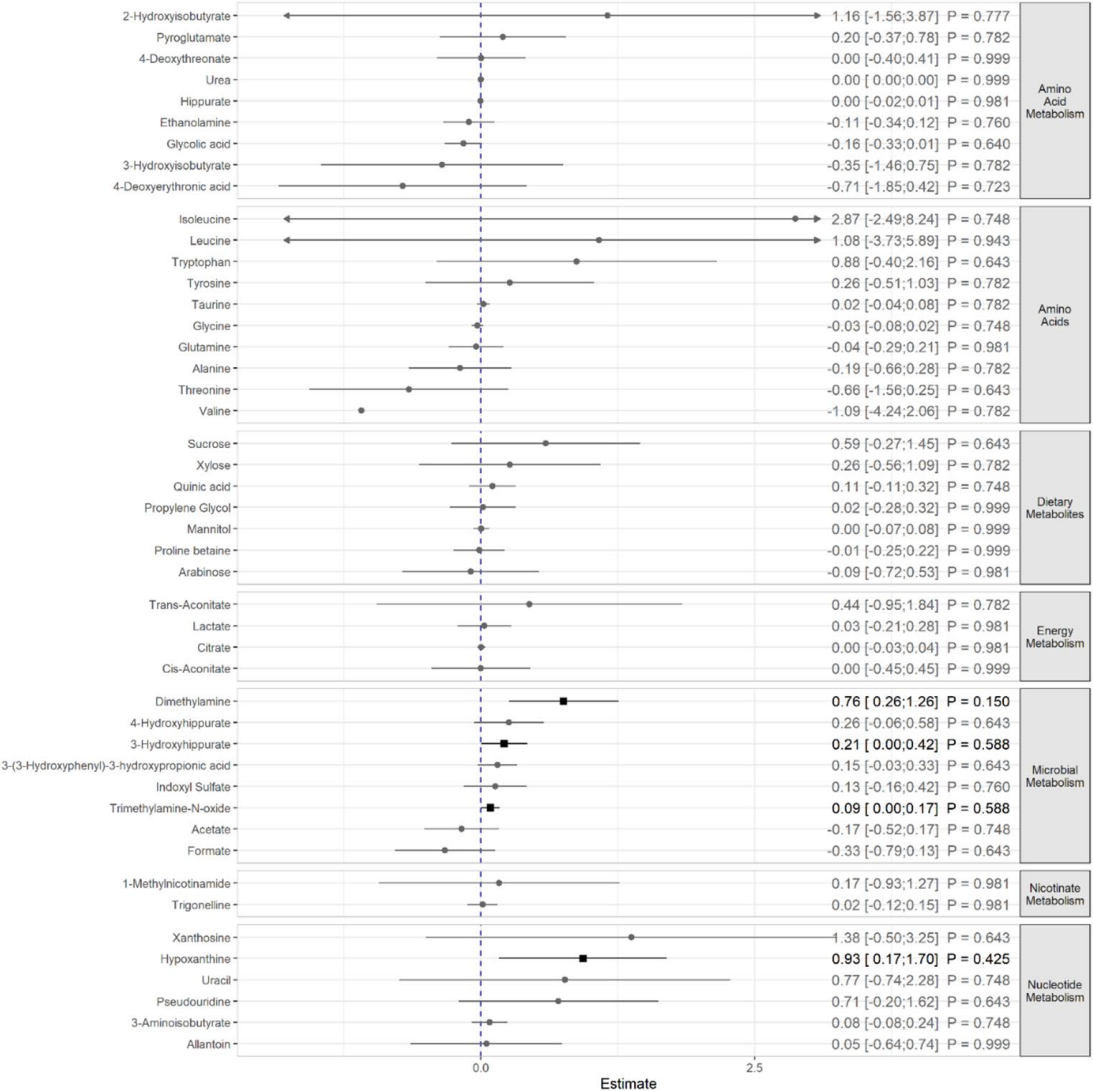
Associations between urinary metabolites and fatigue severity (outcome) adjusted for age, sex, BMI, education, physical activity, smoking, and risky alcohol consumption. *P*-values were corrected for multiple testing; results shown as squares were statistically significant before *p*-value correction. Metabolites were attributed to metabolic pathways

In multivariable linear regression models, four urinary metabolites showed significant associations with fatigue: hypoxanthine (β = 0.932, 95% CI 0.168–1.697, *p* = 0.017), 3-hydroxyhippurate (β = 0.213, 95% CI 0.003–0.424, *p* = 0.047), dimethylamine (β = 0.756, 95% CI 0.255–1.257, *p* = 0.003), and trimethylamine-N-oxide (β = 0.088, 95% CI 0.002–0.174, *p* = 0.045, Supplementary Table S2). However, after correction for multiple comparisons using the Benjamini–Hochberg procedure, none of these associations remained statistically significant (Fig. 2).

After additional adjustment for fatigue-related health conditions, including depression, CVD, diabetes, self-reported history of COVID-19 and cancer, results were consistent with the main model (Supplementary Fig. S1). Before FDR adjustment, hypoxanthine (β = 0.817, 95% CI 0.074–1.560, *p* = 0.031), 3-hydroxyhippurate (β = 0.244, 95% CI 0.040–0.447, *p* = 0.019), dimethylamine (β = 0.726, 95% CI 0.236–1.215, *p* = 0.004), and trimethylamine-N-oxide (β = 0.088, 95% CI 0.006–0.171, *p* = 0.036) were positively associated with fatigue (Supplementary Table S3), but none remained significant after FDR adjustment.

Among the tested interactions between age, sex, and BMI with each urinary metabolite, only the interaction between BMI and hypoxanthine reached statistical significance (*p* = 0.003). No evidence for effect modification by age or sex was observed for any of the investigated associations. Consequently, stratified analyses, adjusted for sociodemographic characteristics and lifestyle factors, were conducted to examine the association between hypoxanthine and fatigue across BMI categories (Table 3). In the “underweight or normal weight” (β = −0.216, 95% CI −0.236–0.804, *p* = 0.677) and “overweight” (β = 0.488, 95% CI −1.303–2.278, *p* = 0.592) groups, no significant associations were observed. In the obese subgroup, each 0.01 mmol/L increase in urinary hypoxanthine was associated with an increase in fatigue severity (β = 2.974, 95% CI 1.550–4.398, *p* < 0.001). These findings remained consistent after additional adjustment for fatigue-related health conditions.

**Table 3:** Association of urinary hypoxanthine with fatigue severity

BMI category	β (95% CI_lower_; 95% CI_upper_)	*p*-value
Underweight & normal	-0.216 (-1.236; 0.804)	0.677
Overweight	0.488 (-1.303; 2.278)	0.592
Obese	2.974 (1.550; 4.398)	< 0.001*

*p < 0.05

Linear regression model adjusted for age, sex, BMI, education level, physical activity level, smoking status, and alcohol consumption

## Discussion

In the present study, urinary concentrations of hypoxanthine, dimethylamine, trimethylamine-N-oxide and 3-hydroxyhippurate were positively associated with fatigue, with the association for hypoxanthine observed only in individuals with obesity. However, these associations did not remain statistically significant after correction for multiple testing. We discuss the observed associations in our main models to provide an exploratory perspective on potential biochemical pathways linked to fatigue. These analyses are hypothesis-generating and aim to guide future studies rather than establish definitive causal mechanisms.

Hypoxanthine is a purine degradation product that accumulates under conditions of increased adenosine triphosphate (ATP) turnover in cellular energy stress (Saugstad, 1988). It is converted to xanthine and uric acid by xanthine oxidoreductase (XOR) in the liver before renal or intestinal excretion, while a large proportion is recycled to ATP through the purine salvage pathway (Murray, 1971).

Several studies have reported altered levels of hypoxanthine and related purine metabolites in fatigue, but overall evidence is limited and most information comes from clinical samples of individuals meeting the ME/CFS diagnosis criteria. Moreover, to the best of our knowledge, no study has investigated urinary metabolite concentrations in ME/CFS patients and controls. Abujrais et al. observed elevated plasma hypoxanthine in 38 ME/CFS patients compared with 24 healthy controls, which aligns with our urinary findings (Abujrais et al., 2024). By contrast, Armstrong et al. reported lower serum hypoxanthine and higher urinary allantoin in 46 ME/CFS patients versus 26 controls (Armstrong et al., 2015), contrasting also with our null finding for allantoin. Also, McGregor et al. found significantly lower serum hypoxanthine concentrations in ME/CFS patients, especially in subgroups with post-exertional malaise (PEM) (McGregor et al., 2019).

Hypoxanthine has been studied as an indicator of low tissue oxygen and energy imbalance (Saugstad, 1988). The BMI-specific association in our study may be attributable to adipose tissue hypoxia (Hosogai et al., 2007), consistent with reports of higher circulating hypoxanthine in individuals with obesity (Furuhashi et al., 2020). Hypoxia impairs mitochondrial oxidative phosphorylation, leading to cellular energy deficiency, and can enhance inflammation via reactive oxygen species, two core features discussed in fatigue pathophysiology (Lacourt et al., 2018; Morris & Maes, 2014). We cannot establish causal mechanisms based on our hypothesis-generating observations. Nonetheless, some studies suggest that purine degradation may serve as a compensatory mechanism to maintain cellular energy charge under limited ATP generation, similar to activation of anaerobic metabolism reflected by elevated lactate levels (Nozaki et al., 2009; Saugstad, 1977).

In our analysis, trimethylamine-N-oxide (TMAO) and its downstream metabolite dimethylamine (DMA) were significantly associated with fatigue before correction for multiple testing. Both are derived from dietary choline and L-carnitine, present in red meat, fish, and dairy products, through gut microbiota-dependent conversion and are mainly excreted in urine (Rath et al., 2017). DMA can be formed either through TMAO demethylase or trimethylamine (TMA) dehydrogenase (Chhibber-Goel et al., 2017) and has received comparatively little scientific attention, likely due to its role as a secondary product (Hsu et al., 2020).

TMAO has frequently been measured in metabolomics studies of ME/CFS and other cohorts, but associations with fatigue have generally not been observed (Che et al., 2022; Nagy-Szakal et al., 2018), except for one study reporting an association in male patients with advanced chronic kidney disease and thus impaired excretion capacity (Massy et al., 2022). This may indicate that changes in bodily TMAO concentrations may be better captured in urine samples rather than blood samples. TMAO is associated with a range of chronic diseases, most notably cardiovascular disease (CVD) and chronic kidney disease (CKD) (Brown & Hazen, 2014; Kim et al., 2016). A large prospective study of over 136,000 Chinese adults showed that higher urinary TMAO levels were associated with an increased risk of incident coronary heart disease (Yu et al., 2019). Elevated TMAO has also been observed in inflammatory bowel disease (IBD), colorectal cancer, cognitive decline (Bae et al., 2014; Brunt et al., 2021; Wilson et al., 2015) and impaired glucose tolerance (Gao et al., 2014). A common feature across these disorders is chronic low-grade inflammation (Kim et al., 2025), which has also been proposed to play a role in fatigue, particularly in ME/CFS (Jonsjö et al., 2020).

Another pathway discussed in TMAO-associated diseases is the gut microbiome, as TMAO levels are largely influenced by microbial enzymatic activity (Romano et al., 2015). Thus, TMAO could be considered as a marker potentially influenced by underlying shifts in gut microbial composition or activity (Cho et al., 2017). In ME/CFS, multiple studies have linked fatigue severity to gut microbiome dysbiosis, including reduced microbial diversity and compositional changes (Jurek & Castro-Marrero, 2024; Naviaux et al., 2016). This aligns with the high prevalence of gastrointestinal symptoms in affected individuals (Steinsvik et al., 2023; Tate et al., 2023) and symptom improvement following microbiome-targeted interventions (Tate et al., 2023). However, no consistent microbial signature has been identified in ME/CFS, highlighting the need to validate promising microbial metabolites such as TMAO (Jurek & Castro-Marrero, 2024; König et al., 2021).

Another metabolite positively associated with fatigue in our study was 3-hydroxyhippurate (3HHA), a glycine-conjugated derivative of microbially derived 3-hydroxybenzoic acid (3HBA) (Buck et al., 1979). Structurally related metabolites, hippurate and 4-hydroxyhippurate (4HHA) are likewise generated by glycine conjugation, however, neither was associated with fatigue in our study (Lees et al., 2013).

3HHA was analyzed in some human metabolomics studies, yet few have linked it to clinical phenotypes, and none to fatigue (Al Hageh et al., 2020; Xiong et al., 2016; Zhao et al., 2010). In a metabolomics study, reduced urinary 3HHA distinguished pre-diabetic subjects from normoglycemic controls alongside other gut microbiota–associated metabolites, including hippurate (Zhao et al., 2010). Hippurate is better studied and correlates with microbiome diversity, with lower levels reported in IBD (Williams et al., 2010) and higher levels associated with increased glucose tolerance and lower risk of metabolic syndrome (Pallister et al., 2017). 3HHA may therefore capture a more specific segment of microbial aromatic acid metabolism, and our exploratory findings in the context of fatigue might point to distinct host–microbial processes not reflected by hippurate, that warrant further investigation.

In summary, all three metabolite groups, purine degradation products, TMAO-related microbial metabolites, and aromatic acid conjugates, are related to biological themes such as impaired cellular energy metabolism, gut microbiome and low-grade inflammation, which have all been repeatedly implicated in fatigue pathophysiology.

### Strengths and limitations

This study has several notable strengths. First, it includes a robust sample size of 570 participants assessed with validated, standardized fatigue and clinical assessment methods. The availability of extensive sociodemographic, lifestyle, and comorbidity data allowed for comprehensive multivariable adjustment. Additionally, the application of metabolomics analyses to urinary samples provided a non-invasive and innovative approach to explore biochemical correlates of fatigue in the general population.

Several limitations should be considered. Due to the cross-sectional design, causal relationships cannot be inferred, and future longitudinal or experimental studies are needed to determine whether metabolites contribute to fatigue or serve as biomarkers of altered energy or microbial metabolism. Besides being less invasive and providing insights into gut microbiome activity, urinary metabolites reflect compounds excreted via renal clearance and therefore offer a targeted perspective that complements blood-based metabolomic analyses. However, compared with the blood metabolome, they primarily represent end products of metabolism and global metabolic profiles rather than acute pathophysiological processes (Zhang et al., 2012), and are more strongly influenced by environmental factors such as diet and kidney function (Walsh et al., 2006). Several metabolites in our study, particularly trimethylamine-N-oxide, dimethylamine, and hippurate-related compounds, are strongly influenced by dietary intake and gut microbiome activity. Although dietary data were collected, they were not included in the regression models, which may confound the observed associations and limits the interpretation of these findings. The interpretation of BMI as a proxy for adiposity should be approached with caution, as it may both underestimate and overestimate true adiposity. However, its use as a surrogate measure at the population level in epidemiological studies remains widely supported (Rubino et al., 2025). The generalizability of our findings is restricted by the regional recruitment area and the age range of the participants, limiting applicability to populations above 75 years. Interindividual variation in microbiome composition, which strongly influences microbial metabolites, may differ across geographic, dietary, and cultural contexts, underscoring the need for replication in more diverse cohorts. Differences across studies may also reflect variation in sampling matrices, timing, clinical context, and interindividual variability in enzymatic activity or renal excretion. Finally, residual confounding by unmeasured factors cannot be ruled out.

## Conclusion

Our findings suggest potential associations between specific urinary metabolites and fatigue in the general population, before adjusting for multiple testing. TMAO, DMA, and 3HHA were positively associated with fatigue in the overall cohort, whereas the association of hypoxanthine was limited to the obese group.

This exploratory analysis provides initial insights into potential urinary metabolite–fatigue associations, but future research should use a longitudinal approach with sufficiently large numbers of participants, incorporate microbiota analyses, and validate these biomarkers in diverse populations to more accurately capture metabolite-fatigue relationships.

## Supplementary Information

Below is the link to the electronic supplementary material.

Supplementary Material 1

## Data Availability

The data underlying this article cannot be shared publicly because the data are subject to national data protection laws and restrictions that were imposed by the ethics committee of the Ludwig-Maximilians University, Munich, to ensure data privacy of the study participants because they did not explicitly consent to the data being made publicly available. The data will be shared at reasonable requests to the corresponding.

## Abbreviations

ATP: Adenosine triphosphate
AUDIT: Alcohol Use Disorders Identification test
BMI: Body mass index
CKD: Chronic kidney disease
CRP: C-reactive protein
CVD: Cardiovascular disease
DMA: Dimethylamine
EHIS-PAQ: European Health Interview Survey-Physical Activity Questionnaire
FAS: Fatigue Assessment Scale
FDR: False discovery rate
3HBA: 3-hydroxybenzoic acid
3HHA: 3-hydroxyhippurate
¹H-NMR: Proton nuclear magnetic resonance spectroscopy
IBD: Inflammatory bowel disease
ISCED: International Standard Classification of Education
ME/CFS: Myalgic encephalomyelitis/chronic fatigue syndrome
MEIA: Metabolism, Nutrition, and Immune System in Augsburg study
PEM: Post-exertional malaise
TMA: Trimethylamine
TMAO: Trimethylamine-N-oxide
XOR: Xanthine oxidoreductase
