# Supplementary material for: Urinary metabolites and fatigue in a population-based metabolomics study: an exploratory analysis
Source: Metabolomics. 2026 Apr 29;22(3):58. doi: 10.1007/s11306-026-02432-6 (PMC13124768; doi:10.1007/s11306-026-02432-6)
Supplement: Supplementary file 1 — Supplementary Material 1 [file 11306_2026_2432_MOESM1_ESM.pdf]

Supplement

Metabolomics

Urinary Metabolites and Fatigue in a Population-Based Metabolomics Study: An exploratory analysis

Annika Kneipp[1], Inge Kirchberger[1], Dennis Freuer[1], Christine Meisinger[1], Jakob Linseisen[1]

[1] *Epidemiology, Faculty of Medicine, University of Augsburg, Augsburg, Germany*

*Corresponding author:
Prof. Dr. Jakob Linseisen
Institute of Epidemiology, University Hospital Augsburg
Stenglinstr. 2
86156 Augsburg,
Germany
Tel.: +49-821-5986471
Email: jakob.linseisen@med.uni-augsburg.de

Table S1 Creatinine-corrected urinary metabolite concentrations [mmol/mmol creatinine x 100] in the total study population and stratified by sex

	**Total (*n* = 570)**	**Female (*n* = 320)**	**Male (*n* = 250)**	***p*-values[a]**
Acetate	0.463 (0.261; 0.794)	0.591 (0.341; 0.977)	0.340 (0.203; 0.597)	< 0.001*
Alanine	1.728 (1.323; 2.215)	1.690 (1.337; 2.162)	1.810 (1.313; 2.404)	0.215
Allantoin	0.543 (0.270; 0.962)	0.467 (0.235; 0.813)	0.674 (0.362; 1.136)	< 0.001*
2-Hydroxyisobutyrate	0.494 (0.401; 0.592)	0.505 (0.398; 0.604)	0.482 (0.406; 0.570)	0.252
Arabinose	0.444 (0.296; 0.645)	0.497 (0.330; 0.721)	0.399 (0.284; 0.553)	< 0.001*
3-Aminoisobutyrate	0.498 (0.160; 1.249)	0.544 (0.187; 1.267)	0.413 (0.155; 1.142)	0.072
3-Hydroxyisobutyrate	0.681 (0.522; 0.872)	0.677 (0.485; 0.873)	0.683 (0.544; 0.867)	0.524
Cis-Aconitate	1.726 (1.386; 2.230)	1.974 (1.476; 2.446)	1.585 (1.244; 1.927)	< 0.001*
Citrate	19.103 (11.615; 28.874)	25.568 (17.421; 33.969)	13.560 (8.370; 19.042)	< 0.001*
Dimethylamine	2.970 (2.705; 3.330)	3.119 (2.867; 3.430)	2.778 (2.558; 3.074)	< 0.001*
4-Deoxyerythronic acid	0.685 (0.529; 0.911)	0.711 (0.525; 0.945)	0.668 (0.546; 0.885)	0.164
4-Deoxythreonate	2.094 (1.554; 2.715)	1.915 (1.418; 2.413)	2.406 (1.812; 3.147)	< 0.001*
Ethanolamine	3.992 (3.014; 5.060)	4.299 (3.184; 5.333)	3.673 (2.818; 4.609)	< 0.001*
Formate	1.509 (0.996; 1.989)	1.604 (1.090; 2.112)	1.354 (0.888; 1.944)	0.005*
Glutamine	2.029 (1.135; 3.161)	2.080 (1.065; 3.276)	1.956 (1.191; 3.035)	0.775
Glycine	8.007 (5.458; 11.543)	9.311 (6.680; 13.833)	6.419 (4.626; 9.022)	< 0.001*
Glycolic acid	3.882 (2.749; 5.242)	3.886 (2.643; 5.273)	3.876 (2.782; 5.106)	0.576
Hippurate	23.768 (14.685; 38.276)	27.845 (17.073; 43.882)	20.077 (11.041; 31.895)	< 0.001*
3-(3-Hydroxyphenyl)-3-hydroxypropionic acid	1.412 (0.469; 2.891)	1.710 (0.555; 3.531)	1.203 (0.303; 2.541)	< 0.001*
Hypoxanthine	0.824 (0.592; 1.125)	0.880 (0.611; 1.163)	0.758 (0.588; 1.053)	0.010*
Isoleucine	0.086 (0.056; 0.137)	0.100 (0.061; 0.159)	0.073 (0.053; 0.108)	< 0.001*
Indoxyl Sulfate	2.528 (1.792; 3.478)	2.766 (1.970; 3.707)	2.251 (1.689; 3.134)	< 0.001*
Lactate	0.893 (0.583; 1.523)	1.256 (0.827; 2.177)	0.653 (0.419; 0.883)	< 0.001*
Leucine	0.172 (0.134; 0.224)	0.170 (0.129; 0.227)	0.174 (0.139; 0.212)	0.601
Mannitol	1.045 (0.405; 3.199)	1.105 (0.388; 3.511)	0.958 (0.430; 2.912)	0.551
3-Hydroxyhippurate	1.409 (0.721; 2.817)	1.536 (0.767; 3.112)	1.238 (0.575; 2.402)	0.023*
1-Methylnicotinamide	0.619 (0.457; 0.843)	0.690 (0.508; 0.950)	0.550 (0.416; 0.748)	< 0.001*
Pyroglutamate	2.252 (1.907; 2.667)	2.350 (1.993; 2.733)	2.077 (1.815; 2.519)	< 0.001*
4-Hydroxyhippurate	1.047 (0.731; 1.557)	1.148 (0.792; 1.637)	0.951 (0.679; 1.402)	< 0.001*
Propylene glycol	0.362 (0.187; 0.611)	0.384 (0.201; 0.604)	0.331 (0.165; 0.632)	0.141
Proline betaine	0.630 (0.297; 1.491)	0.660 (0.306; 1.582)	0.586 (0.273; 1.437)	0.455
Pseudouridine	3.024 (2.738; 3.321)	3.182 (2.926; 3.445)	2.797 (2.550; 3.060)	< 0.001*

Quinic acid	2.215 (1.028; 3.772)	2.625 (1.212; 4.154)	1.796 (0.800; 2.858)	< 0.001*
Sucrose	0.050 (0.000; 0.227)	0.054 (0.000; 0.243)	0.038 (0.002; 0.199)	0.370
Trans-Aconitate	0.431 (0.321; 0.546)	0.423 (0.311; 0.539)	0.437 (0.335; 0.552)	0.448
Taurine	2.912 (0.571; 6.524)	1.831 (0.175; 5.605)	3.713 (1.473; 6.998)	< 0.001*
Threonine	0.583 (0.390; 0.833)	0.566 (0.360; 0.830)	0.615 (0.410; 0.836)	0.239
Trimethylamine-N-oxide	3.546 (2.480; 5.201)	3.661 (2.563; 5.331)	3.237 (2.383; 4.960)	0.053
Trigonelline	2.851 (1.393; 4.668)	3.481 (1.811; 5.411)	2.122 (0.962; 3.743)	< 0.001*
Tryptophan	0.559 (0.405; 0.736)	0.567 (0.413; 0.770)	0.543 (0.383; 0.703)	0.086
Tyrosine	0.926 (0.649; 1.274)	0.879 (0.609; 1.223)	1.003 (0.736; 1.336)	0.007*
Uracil	0.518 (0.389; 0.677)	0.560 (0.440; 0.759)	0.462 (0.350; 0.611)	< 0.001*
Urea	3211.916 (2387.135; 4121.429)	3326.227 (2438.941; 4392.547)	3054.275 (2364.954; 3875.980)	0.014*
Valine	0.216 (0.166; 0.273)	0.226 (0.182; 0.286)	0.204 (0.151; 0.265)	0.001*
Xanthosine	0.887 (0.783; 1.019)	0.953 (0.839; 1.071)	0.819 (0.748; 0.931)	< 0.001*
Xylose	0.600 (0.353; 0.799)	0.641 (0.397; 0.854)	0.556 (0.315; 0.770)	0.005*

[a] Mann-Whitney-U-Test

Table S2 Association of urinary metabolites with fatigue severity (main regression model)

	**β (95% CI$_{lower}$; 95% CI$_{upper}$)**	***p*-value**
Acetate	-0.175 (-0.517; 0.166)	0.314
Alanine	-0.191 (-0.659; 0.277)	0.423
Allantoin	0.051 (-0.641; 0.743)	0.885
2-Hydroxyisobutyrate	1.156 (-1.560; 3.872)	0.404
Arabinose	-0.092 (-0.717; 0.532)	0.772
3-Aminoisobutyrate	0.081 (-0.082; 0.243)	0.329
3-Hydroxyisobutyrate	-0.353 (-1.460; 0.754)	0.531
Cis-Aconitate	0.000 (-0.451; 0.452)	0.999
Citrate	0.004 (-0.032; 0.040)	0.823
Dimethylamine	0.756 (0.255; 1.257)	0.003*
4-Deoxyerythronic acid	-0.714 (-1.848; 0.421)	0.217
4-Deoxythreonate	0.004 (-0.399; 0.408)	0.983
Ethanolamine	-0.110 (-0.343; 0.124)	0.356
Formate	-0.328 (-0.787; 0.131)	0.161
Glutamine	-0.043 (-0.294; 0.208)	0.736
Glycine	-0.031 (-0.084; 0.022)	0.257
Glycolic acid	-0.159 (-0.328; 0.009)	0.064
Hippurate	-0.002 (-0.018; 0.014)	0.815
3-(3-Hydroxyphenyl)-3-hydroxypropionic acid	0.152 (-0.028; 0.332)	0.097

Hypoxanthine	0.932 (0.168; 1.697)	0.017*
Isoleucine	2.873 (-2.493; 8.239)	0.293
Indoxyl Sulfate	0.132 (-0.155; 0.420)	0.365
Lactate	0.031 (-0.214; 0.275)	0.805
Leucine	1.078 (-3.733; 5.888)	0.660
Mannitol	0.004 (-0.069; 0.077)	0.920
3-Hydroxyhippurate	0.213 (0.003; 0.424)	0.047*
1-Methylnicotinamide	0.168 (-0.933; 1.268)	0.765
Pyroglutamate	0.202 (-0.374; 0.778)	0.492
4-Hydroxyhippurate	0.256 (-0.063; 0.575)	0.115
Propylene glycol	0.019 (-0.282; 0.320)	0.900
Proline betaine	-0.014 (-0.248; 0.220)	0.907
Pseudouridine	0.709 (-0.203; 1.622)	0.127
Quinic acid	0.107 (-0.106; 0.321)	0.324
Sucrose	0.593 (-0.267; 1.453)	0.176
Trans-Aconitate	0.443 (-0.951; 1.838)	0.532
Taurine	0.022 (-0.038; 0.082)	0.470
Threonine	-0.656 (-1.565; 0.254)	0.157
Trimethylamine-N-oxide	0.088 (0.002; 0.174)	0.045*
Trigonelline	0.016 (-0.123; 0.154)	0.824
Tryptophan	0.876 (-0.404; 2.156)	0.180
Tyrosine	0.264 (-0.506; 1.034)	0.501
Uracil	0.769 (-0.741; 2.279)	0.318
Urea	0.000 (-0.000; 0.000)	0.981
Valine	-1.090 (-4.242; 2.061)	0.497
Xanthosine	1.377 (-0.500; 3.254)	0.150
Xylose	0.265 (-0.563; 1.094)	0.529

Linear regression model adjusted for age, sex, BMI, education level, physical activity level, smoking status and alcohol consumption, before FDR correction.

Table S3 Association of urinary metabolites with fatigue severity (extended regression model)

	**β (95% CI_{lower}; 95% CI_{upper})**	***p*-value**
Acetate	-0.096 (-0.427; 0.234)	0.567
Alanine	-0.223 (-0.677; 0.231)	0.335
Allantoin	0.074 (-0.593; 0.742)	0.827
2-Hydroxyisobutyrate	1.527 (-1.156; 4.210)	0.264
Arabinose	0.036 (-0.577; 0.649)	0.908
3-Aminoisobutyrate	0.066 (-0.092; 0.224)	0.409
3-Hydroxyisobutyrate	-0.441 (-1.514; 0.632)	0.420
Cis-Aconitate	0.043 (-0.396; 0.483)	0.846

Citrate	-0.000 (-0.036; 0.036)	0.991
Dimethylamine	0.726 (0.236; 1.215)	0.004*
4-Deoxyerythronic acid	-0.926 (-2.028; 0.177)	0.100
4-Deoxythreonate	0.016 (-0.376; 0.408)	0.935
Ethanolamine	-0.059 (-0.289; 0.171)	0.612
Formate	-0.211 (-0.663; 0.242)	0.361
Glutamine	-0.019 (-0.265; 0.227)	0.879
Glycine	-0.026 (-0.079; 0.026)	0.324
Glycolic acid	-0.145 (-0.309; 0.019)	0.083
Hippurate	-0.002 (-0.017; 0.014)	0.808
3-(3-Hydroxyphenyl)-3-hydroxypropionic acid	0.167 (-0.006; 0.341)	0.059
Hypoxanthine	0.817 (0.074; 1.560)	0.031*
Isoleucine	4.564 (-0.613; 9.741)	0.084
Indoxyl Sulfate	-0.010 (-0.292; 0.272)	0.946
Lactate	0.040 (-0.197; 0.277)	0.739
Leucine	0.341 (-4.318; 5.000)	0.886
Mannitol	0.001 (-0.069; 0.072)	0.973
3-Hydroxyhippurate	0.244 (0.040; 0.447)	0.019*
1-Methylnicotinamide	0.144 (-0.916; 1.204)	0.790
Pyroglutamate	0.184 (-0.374; 0.742)	0.518
4-Hydroxyhippurate	0.267 (-0.042; 0.575)	0.090
Propylene glycol	0.071 (-0.221; 0.362)	0.635
Proline betaine	-0.035 (-0.264; 0.193)	0.760
Pseudouridine	0.602 (-0.301; 1.505)	0.191
Quinic acid	0.123 (-0.083; 0.330)	0.241
Sucrose	0.787 (-0.042; 1.617)	0.063
Trans-Aconitate	0.339 (-1.003; 1.682)	0.620
Taurine	0.032 (-0.026; 0.090)	0.273
Threonine	-0.568 (-1.464; 0.329)	0.214
Trimethylamine-N-oxide	0.088 (0.006; 0.171)	0.036*
Trigonelline	0.020 (-0.114; 0.154)	0.769
Tryptophan	0.969 (-0.269; 2.207)	0.125
Tyrosine	0.352 (-0.395; 1.099)	0.355
Uracil	0.902 (-0.577; 2.380)	0.231
Urea	0.000 (-0.000; 0.000)	0.710
Valine	-0.431 (-3.468; 2.606)	0.780
Xanthosine	1.465 (-0.379; 3.310)	0.119
Xylose	0.314 (-0.489; 1.117)	0.443

Linear regression model adjusted for age, sex, BMI, education level, physical activity level, smoking status, alcohol consumption and additionally for fatigue-related health conditions, including depression, CVD, diabetes, history of COVID-19 and cancer, before FDR correction.

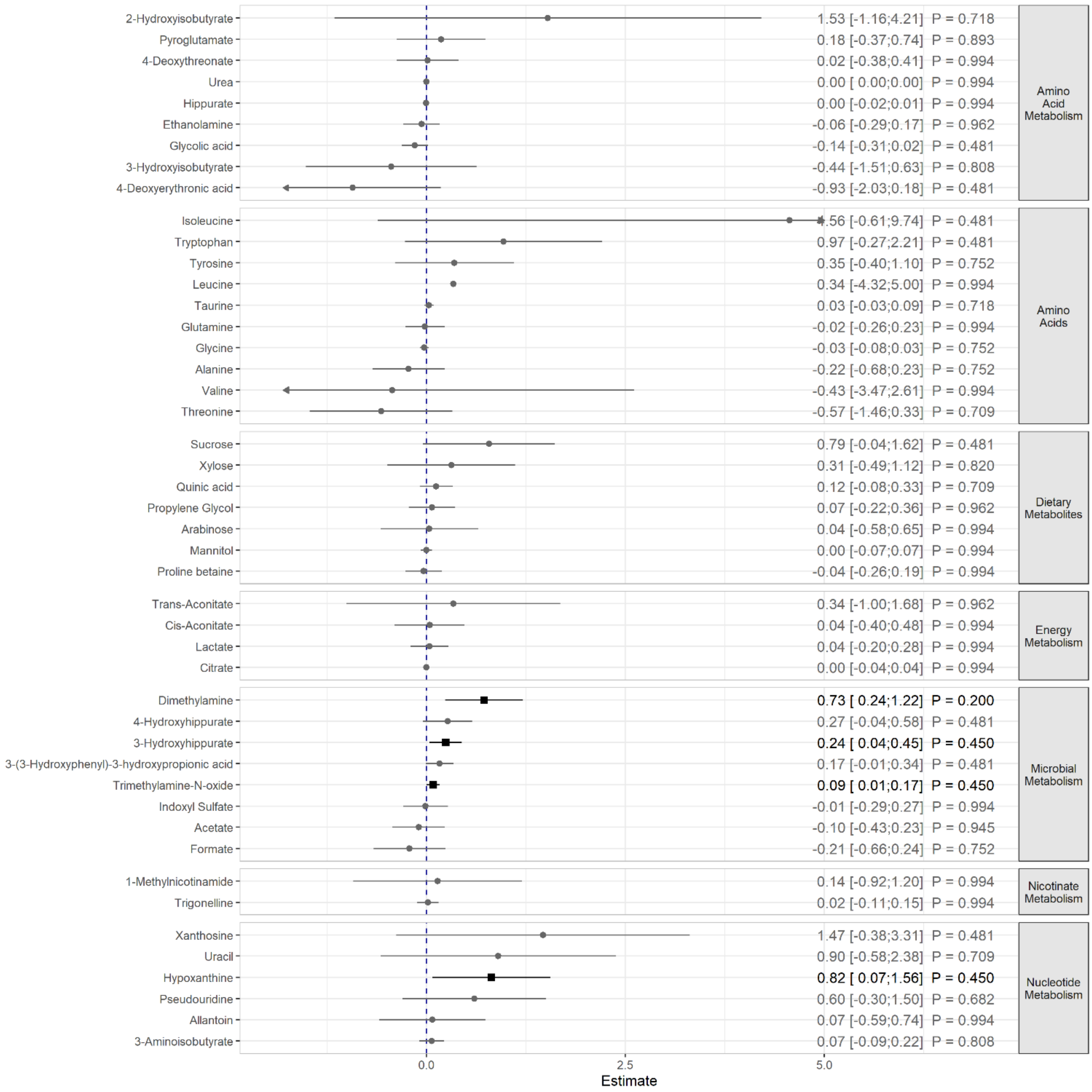

Fig. S1 Associations between urinary metabolites and fatigue severity, with additional adjustment for fatigue-related health conditions (extended regression model). *P*-values were corrected for multiple testing; results shown as squares were statistically significant before *p*-value correction. Metabolites were attributed to metabolic pathways
